# Relevance of Molecular Profiling in Patients With Low-Grade Endometrial Cancer

**DOI:** 10.1001/jamanetworkopen.2022.47372

**Published:** 2022-12-16

**Authors:** Stephanie W. Vrede, Jenneke Kasius, Johan Bulten, Steven Teerenstra, Jutta Huvila, Eva Colas, Antonio Gil-Moreno, Dorry Boll, Maria Caroline Vos, Anne M. van Altena, Jasmin Asberger, Sanne Sweegers, Willem Jan van Weelden, Louis J. M. van der Putten, Frédéric Amant, Nicole C. M. Visser, Marc P. L. M. Snijders, Heidi V. N. Küsters-Vandevelde, Roy Kruitwagen, Xavier Matias-Guiu, Vit Weinberger, Casper Reijnen, Johanna M. A. Pijnenborg

**Affiliations:** 1 Department of Obstetrics and Gynecology, Radboud University Medical Center, Nijmegen, the Netherlands; 2 Department of Obstetrics and Gynecology, Canisius-Wilhelmina Hospital, Nijmegen, the Netherlands; 3 Department of Gynecologic Oncology, Amsterdam Medical Centers and Center of Gynecologic Oncology Amsterdam, Amsterdam, the Netherlands; 4 Department of Pathology, Radboud University Medical Center, Nijmegen, the Netherlands; 5 Department of Health Evidence, Radboud University Medial Center, Nijmegen, the Netherlands; 6 Department of Pathology, University of Turku, Turku, Finland; 7 Biomedical Research Group in Gynecology, Vall Hebron Institute of Research, Universitat Autònoma de Barcelona, Centro de Investigación Biomédica en Red Cáncer, Barcelona, Spain; 8 Gynecological Department, Vall Hebron University Hospital, Centro de Investigación Biomédica en Red Cáncer, Barcelona, Spain; 9 Pathology Department, Vall Hebron University Hospital, Centro de Investigación Biomédica en Red Cáncer, Barcelona, Spain; 10 Department of Obstetrics and Gynecology, Catharina Hospital Eindhoven, the Netherlands; 11 Departement of Obstetrics and Gynecology, Elisabeth-Tweesteden Hospital, the Netherlands; 12 Department of Obstetrics and Gynecology, Medical Center–University of Freiburg, Freiburg, Germany; 13 Department of Oncology, KU Leuven, Leuven, Belgium; 14 Department of Gynaecologic Oncology, Netherlands Cancer Institute and Amsterdam Medical Centers, Amsterdam, the Netherlands; 15 Department of Pathology, Stichting Laboratory for Pathology and Medical Microbiology, Eindhoven, the Netherlands; 16 Department of Pathology, Canisius Wilhelmina Hospital, Nijmegen, the Netherlands; 17 Department of Obstetrics and Gynecology, School for Oncology and Reproduction, Maastricht University Medical Center, Maastricht, the Netherlands; 18 Department of Pathology and Molecular Genetics and Research Laboratory, Hospital Universitari Arnau de Vilanova, University of Lleida, Institut de Recerca Biomèdica de Lleida, Centro de Investigación Biomédica en Red Cáncer, Lleida, Spain; 19 Department of Obstetrics and Gynecology, University Hospital in Brno and Masaryk University, Brno, Czechia; 20 Department of Radiation Oncology, Radboud University Medical Center, Nijmegen, the Netherlands

## Abstract

**Importance:**

Patients with low-grade (ie, grade 1-2) endometrial cancer (EC) are characterized by their favorable prognosis compared with patients with high-grade (ie, grade 3) EC. With the implementation of molecular profiling, the prognostic relevance of tumor grading might lose attention. As most patients present with low-grade EC and have an excellent outcome, the value of molecular profiling for these patients is unclear.

**Objective:**

To determine the association of molecular profiling with outcomes among patients with low-grade EC.

**Design, Setting, and Participants:**

This retrospective cohort study included a multicenter international European cohort of patients diagnosed with EC between 1994 and 2018, with a median follow-up of 5.9 years. Molecular subgroups were determined by next-generation sequencing using single-molecule molecular inversion probes and by immunohistochemistry. Subsequently, tumors were classified as polymerase epsilon (*POLE)-*altered, microsatellite instable (MSI), tumor protein p53 (*TP53)-*altered, or no specific molecular profile (NSMP). Patients diagnosed with any histological subtypes and FIGO (International Federation of Gynecology and Obstetrics) stages of EC were included, but patients with early-stage EC (FIGO I-II) were only included if they had known lymph node status. Data were analyzed February 20 to June 16, 2022.

**Exposures:**

Molecular testing of the 4 molecular subgroups.

**Main Outcomes and Measures:**

The main outcome was disease-specific survival (DSS) within the molecular subgroups.

**Results:**

A total of 393 patients with EC were included, with a median (range) age of 64.0 (31.0-86.0) years and median (range) body mass index (BMI; calculated as weight in kilograms divided by height in meters squared) of 29.1 (18.0-58.3). Most patients presented with early-stage (290 patients [73.8%]) and low-grade (209 patients [53.2%]) disease. Of all patients, 33 (8.4%) had *POLE-*altered EC, 78 (19.8%) had MSI EC, 72 (18.3%) had *TP53*-altered EC, and 210 (53.4%) had NSMP EC. Across all molecular subgroups, patients with low-grade EC had superior 5-year DSS compared with those with high-grade EC, varying between 90% to 100% vs 41% to 90% (*P* < .001). Multivariable analysis in the entire cohort including age, tumor grade, FIGO stage, lymphovascular space invasion, and the molecular subgroups as covariates found that only high-grade (hazard ratio [HR], 4.29; 95% CI, 2.15-8.53; *P* < .001), *TP53*-altered (HR, 1.76; 95% CI, 1.04-2.95; *P* = .03), and FIGO stage III or IV (HR, 4.26; 95% CI, 2.50-7.26; *P* < .001) disease were independently associated with reduced DSS.

**Conclusions and Relevance:**

This cohort study found that patients with low-grade EC had an excellent prognosis independent of molecular subgroup. These findings do not support routine molecular profiling in patients with low-grade EC, and they demonstrate the importance of primary diagnostic tumor grading and selective profiling in low-grade EC to increase cost-effectiveness.

## Key Points

**Question:**

Is tumor molecular profile associated with outcomes among patients with low-grade endometrial cancer?

**Findings:**

In this retrospective multicenter cohort study of 393 patients, outcomes for patients with low-grade endometrial cancer were not associated with molecular subgroup.

**Meaning:**

These findings do not support routine molecular profiling in patients with low-grade endometrial cancer.

## Teaser

This cohort study assesses the association of molecular profiling with disease-specific survival among patients with low-grade endometrial cancer.

## Introduction

More than 85% of patients with endometrial cancer (EC) present with low-grade histology (ie, grade 1-2) and International Federation of Gynecology and Obstetrics (FIGO) early-stage (ie, I-II) endometrioid EC and have a favorable prognosis, with a 5-year overall survival of 95%.^1,2^ Standard treatment is hysterectomy with bilateral salpingo-oophorectomy, including lymph node staging for patients with substantial risk of lymph node metastasis.^2^

The Cancer Genome Atlas defined 4 important prognostic molecular subgroups in EC based on integrated genomic data: ultramutated tumors with polymerase epsilon (*POLE*; OMIM 174762) alteration, microsatellite instability (MSI), copy-number-high with frequent tumor protein p53 (*TP53*; OMIM 191170) alteration, and copy-number-low (also known as no specific molecular profile [NSMP]). These subgroups increase insight in biological tumor behavior based on molecular signature beyond current morphological classification.^3^ Patients with *TP53*-altered tumors have the worst outcome, representing 15% of all EC diagnoses and responsible for 50% to 70% of all EC-related mortality.^4,5^

For decades, tumor grading and FIGO staging have been used to guide primary and adjuvant treatment.^6^ Currently, with incorporation of the molecular classification to guide adjuvant treatment, the prognostic relevance of tumor grading has gained less attention.^7^ Molecular profiling has been shown to improve prognostication mainly in patients with high-grade EC, probably due to poor interobserver reproducibility of morphological classification and the prognostic and intratumoral heterogeneity of high-grade ECs.^5,8^ To our knowledge, no data have been reported about the prognostic relevance of molecular profiling specifically in patients with low-grade EC. The aim of this study is to determine the prognostic relevance of molecular profiling within low-grade EC. As most patients present with low-grade EC and have an excellent outcome, we hypothesized that molecular profiling might be less useful in these patients.

## Methods

This cohort study was approved by the institutional review board of Radboud University Medical Center and the institutional review boards of all participating centers. Data used in this study were from previous published studies by our research group; therefore, informed consent was waived for participants. This study followed the Strengthening the Reporting of Observational Studies in Epidemiology (STROBE) reporting guideline.

### Data Source

This retrospective European multicenter study used data from 4 previously published studies^9,10,11,12^ and 1 study that has not been published yet, all published by our research group. A baseline overview and flowchart of the included studies are shown in eTable 1 and eFigure 1 in Supplement 1.

### Patients

All patients were surgically treated between 1994 and 2018 (median, 2006). Inclusion criteria for this study were: patients diagnosed with primary EC with all histological subtypes and FIGO stages, with available EC tissue samples, from which tumors were successfully classified according molecular profiling or the Proactive Molecular Risk Classifier for Endometrial Cancer^13^ classification. The exclusion criterion was unknown lymph node status in FIGO early-stage disease.

**Figure 1.**
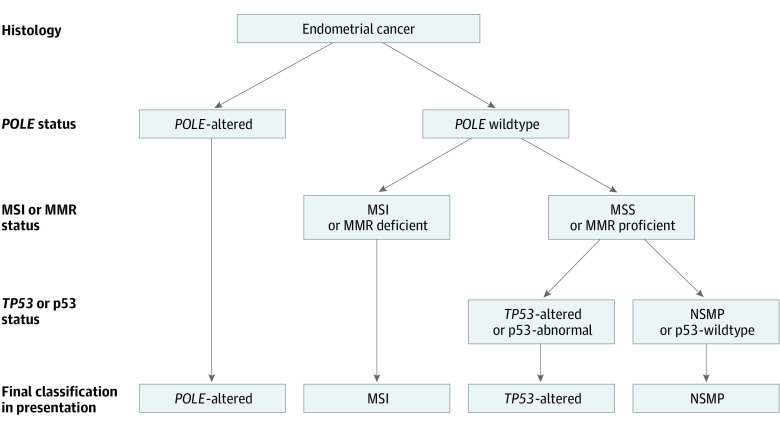
Diagnostic Algorithm of Patients Diagnosed With Molecular Profiling or With Immunohistochemistry MMR indicates mismatch repair protein; MSI, microsatellite instability; and NSMP, no specific molecular profile.

Patients were classified into 1 of 4 molecular subgroups according to the diagnostic algorithm (Figure 1): *POLE-*altered, MSI, *TP53*-altered, and NSMP. Multiple-classifiers were classified as the molecular subgroup with the best prognosis.^14^

### DNA Analysis

Representative areas of EC in the surgical specimen were marked and selected for formalin-fixed paraffin-embedded 20-μm thick sections. Slides were cut from these formalin-fixed paraffin-embedded sections and stained with hematoxylin and eosin. Tumor areas were marked on these slides, and the tumor cell percentage was estimated. These specimens were digested overnight at 56 °C in TET-lysis buffer (10 mmol/L Tris/hydrochloride, pH 8.5; 1 mmol/L ethylenediaminetetraacetic acid, pH 8.0; and 0.01% polysorbate 20 [Tween-20; Thermo Fisher]) with 5% Chelex-100 (Bio-Rad) and 0.2% proteinase K, with subsequent inactivation at 95 °C for 10 minutes. After this was centrifugated, the supernatant was transferred into a clean tube. DNA concentration was determined using the Qubit Broad Range Kit (Thermo Fisher Scientific).

### Single-Molecule Molecular Inversion Probe Design and Library Preparation

Samples were analyzed with single-molecule molecular inversion probes (smMIPs). The design (Integrated DNA Technologies), as well as the library preparation, were previously published.^15^ Further detailed information on smMIP design, library preparation, and sequencing are provided in the eMethods in Supplement 1.

### Immunohistochemical Staining and Scoring

Detailed information about the immunohistochemical staining for p53 and mismatch repair endonucleases PMS2 and MSH6 can be found in the eMethods in Supplement 1 and original published studies.^9,10^ In brief, staining for p53 was considered outside reference range when more than 80% of tumor cell nuclei showed strong expression (overexpression) or when there was complete absence of nuclear staining (null expression). Mismatch repair deficiency was defined as total loss of nuclear staining of PMS2 or MSH6 in the presence of a positive internal control.

### Disease Classification

Early-stage disease was defined as FIGO stage I or II, and advanced-stage was defined as FIGO III or IV. Low-grade EC was defined as grade 1 and grade 2 EC, and high-grade EC was defined as grade 3 endometrioid EC and nonendometrioid EC, according to the latest European Society of Gynaecological Oncology, European Society for Radiotherapy and Oncology, and European Society of Pathology and World Health Organization guidelines.^2,16^ The included patients in our retrospective cohort received either full lymphadenectomy or no lymphadenectomy, as sentinel lymph node procedure was not routinely incorporated yet.

### Statistical Analysis

Statistical analyses were performed on SPSS version 25.0 (IBM) using χ^2^, Fisher exact test, Mann-Whitney *U* test, Kaplan-Meier survival analysis, and univariable and multivariable Cox regression analysis. For survival curves, including Hall-Wellner confidence bands, we used SAS version 9.4 (SAS Institute). Two-tailed *P* < .05 was considered statistically significant. The assumption of proportionality for the included variables was tested with log-minus-log curves and time-dependent covariate (time × covariate). Disease-specific survival (DSS) was defined as time from date of surgery to date of death from EC, all censored by date of last contact. We validated our data with the open access database of Kandoth et al^3^ by performing Kaplan-Meier analysis. Method and baseline characteristics can be found in the original article.^3^ Data were analyzed February 20 to June 16, 2022.

## Results

### Patients

In total, 689 patients were available with successful DNA analysis, of whom 296 (42.9%) were excluded based on unknown lymph node status in FIGO early-stage disease (eFigure 1 in Supplement 1)*.* Baseline characteristics of the included vs excluded patients are shown in eTable 2 in Supplement 1. Of 393 included patients, median (range) age was 64.0 (31.0-86.0) years, and median (range) body mass index (calculated as weight in kilograms divided by height in meters squared) was 29.1 (18.0-58.3) (Table 1). Baseline characteristics of the included patients according to the 4 molecular subgroups are shown in Table 1. Molecular subgroup distribution was 33 patients (8.4%) with *POLE*-altered disease, 78 patients (19.8%) with MSI disease, 72 patients (18.3%) with *TP53*-altered tumors, and 210 patients (53.4%) with NSMP. Low- and high-grade EC were equally distributed in patients with *POLE-*altered and MSI tumors. Most patients with *TP53*-altered tumors had high-grade EC, and most NSMP tumors were low-grade EC (Table 1). The EC-related mortality was highest in the *TP53*-altered subgroup (33 patients [45.8%]) compared with the other molecular subgroups (NSMP: 33 patients [15.7%], MSI: 6 patients [7.7%]; *POLE*-altered: 1 patients [3.0%]). Excluded patients had similarly favorable DSS outcomes for all molecular subgroups within low-grade EC.

**Table 1.** Baseline Characteristics of the Included Study Cohort According to the 4 Molecular Subgroups

Characteristic	Patients by molecular subtype, No. (%)					*P* value
	Total (N = 393)	*POLE*-alt (n = 33)	MSI (n = 78)	*TP53*-alt (n = 72)	NSMP (n = 210)	
**Patient characteristics**						
Age, median (range), y	64.0 (31.0-86.0)	58.0 (31.0-78.0)	65.0 (43.0-83.0)	64.5 (35.0-82.0)	63.5 (37.0-86.0)	.001
BMI, median (range)	29.1 (18.0-58.3)	31.3 (18.4-58.3)	29.5 (21.90-46.9)	31.2 (21.2-41.1)	27.0 (18.0-38.9)	.004
**Primary treatment**						
Lymph node dissection						
No	12 (3.1)	0	3 (3.8)	2 (2.8)	7 (3.3)	.26
Yes	376 (95.7)	33 (100)	75 (96.2)	67 (93.1)	201 (95.7)	
Pelvic	214 (56.9)	21 (63.6)	47 (62.7)	25 (37.3)	121 (60.2)	
Para-aortic	13 (3.4)	0	1 (1.3)	4 (6.0)	8 (4.0)	
Pelvic and para-aortic	54 (14.4)	4 (12.1)	8 (10.7)	12 (17.9)	30 (14.9)	
Unknown which nodes	95 (25.3)	8 (24.2)	19 (25.3)	26 (38.8)	42 (20.9)	
Unknown	5 (1.3)	0	0	3 (4.2)	2 (1.0)	
**Final pathologic characteristics**						
Histology						
EEC	318 (80.9)	28 (84.8)	69 (88.5)	41 (56.9)	180 (85.7)	<.001
Non-EEC	75 (19.1)	5 (15.2)	9 (11.5)	31 (43.1)	30 (14.3)	
Grade						
1-2	209 (53.2)	17 (51.5)	41 (52.6)	13 (18.1)	138 (65.7)	<.001
3	184 (46.8)	16 (48.5)	37 (47.4)	59 (81.9)	72 (34.3)	
Myometrial invasion						
<50%	197 (50.1)	13 (39.4)	42 (53.8)	32 (44.4)	110 (52.4)	.14
>50%	194 (49.4)	19 (57.6)	35 (44.9)	40 (55.6)	100 (47.6)	
Unknown	2 (0.5)	1 (3.0)	1 (1.3)	0	0	
LVSI						
No	304 (77.4)	27 (81.8)	64 (82.1)	41 (56.9)	172 (81.9)	<.001
Yes	89 (22.6)	6 (18.2)	14 (17.9)	31 (43.1)	38 (18.1)	
Lymph nodes						
N0	305 (77.6)	29 (87.9)	68 (87.2)	46 (63.9)	162 (77.1)	.02
N1	43 (10.9)	1 (3.0)	5 (6.4)	13 (18.1)	24 (11.4)	
Pelvic	18 (41.9)	1 (100)	2 (40.0)	6 (46.2)	9 (37.5)	
Para aortic	7 (16.3)	0	0	4 (30.8)	3 (12.5)	
Pelvic and para-aortic	6 (13.9)	0	2 (40.0)	0	4 (16.7)	
Unknown which nodes	12 (27.9)	0	1 (20.0)	3 (23.0)	8 (33.3)	
No information	40 (10.2)	3 (9.1)	5 (6.4)	13 (18.1)	24 (11.4)	
FIGO stage						
Early (I-II)	290 (73.8)	27 (81.8)	68 (87.2)	37 (51.4)	158 (75.2)	<.001
Advanced (III-IV)	103 (26.2)	6 (18.2)	10 (12.8)	35 (48.6)	52 (24.8)	
Adjuvant treatment						
None	97 (24.7)	6 (18.2)	15 (19.2)	17 (23.6)	59 (28.1)	.02
Radiotherapy	225 (57.3)	20 (60.6)	56 (71.8)	34 (47.2)	115 (54.8)	
EBRT	67 (29.8)	8 (40.0)	15 (26.8)	16 (47.1)	28 (24.3)	
VBT	89 (39.6)	6 (30.0)	25 (44.6)	7 (20.6)	7 (20.6)	
ERBT+VBT	47 (20.9)	5 (25.0)	10 (17.9)	5 (14.7)	5 (14.7)	
Unknown	22 (9.8)	1 (5.0)	6 (10.7)	6 (17.6)	6 (17.6)	
Chemotherapy	33 (8.4)	2 (6.1)	2 (2.6)	13 (18.1)	16 (7.6)	
Chemoradiation	34 (8.7)	5 (15.2)	4 (5.1)	6 (8.3)	19 (9.0)	
Unknown	4 (1.0)	0	1 (1.3)	2 (2.8)	1 (0.5)	
Mortality						
Recurrence	74 (18.8)	1 (3.1)	12 (15.8)	30 (50.8)	31 (15.4)	<.001
Mortality	90 (22.9)	2 (6.1)	8 (10.3)	38 (52.8)	42 (20.0)	<.001
EC-related mortality	73 (18.6)	1 (3.0)	6 (7.7)	33 (45.8)	33 (15.7)	<.001

Abbreviations: alt, altered; EBRT, external beam radiation therapy; EC, endometrial cancer; EEC, endometrioid endometrial cancer; FIGO, Federation International of Gynecology and Obstetrics; LVSI, lymphovascular space invasion; MSI, microsatellite instability; NSMP, No specific molecular profile; VBT, vaginal brachytherapy.

### Outcome

For the independent variables in Cox regression models, the proportional hazard assumption was checked. Results of testing the proportional hazard assumption show that all the variables were satisfied.

**Figure 2.**
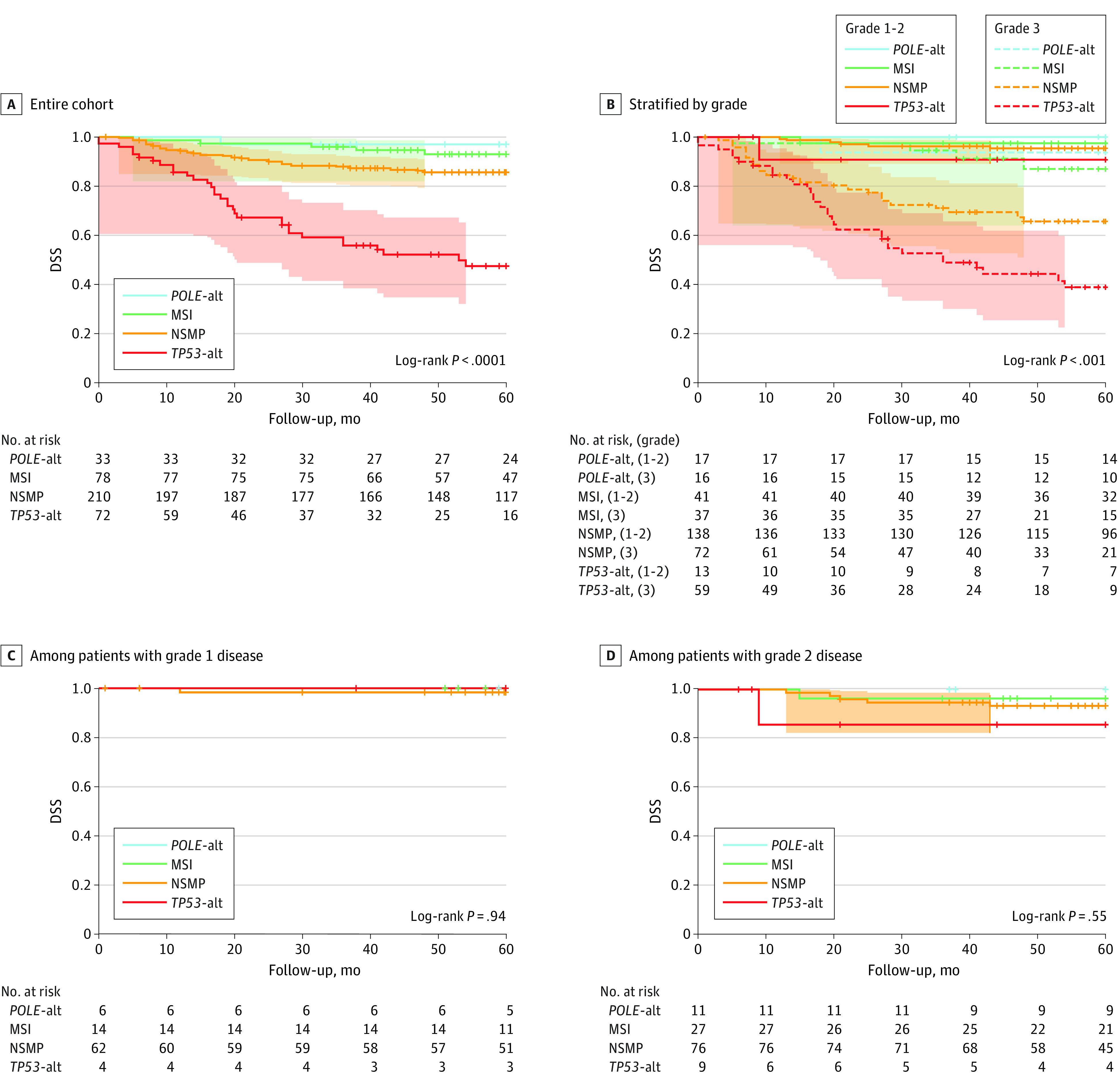
Five-Year Disease-Specific Survival in Patients with Endometrial Cancer in the Entire Cohort and by Molecular Subtype and Grade Alt indicates altered; MSI, microsatellite instability; NSMP, no specific molecular profile.

The 5-year DSS of the included study cohort was worst for *TP53*-altered tumors and best for *POLE-*altered tumors (Figure 2A). Across all molecular subgroups, patients with low-grade EC had an outstanding 5-year DSS compared with patients with high-grade EC, varying between 90% to 100% vs 41% to 90% (*P* < .001) (Figure 2B). For all the molecular subgroups in patients with grade 1 EC, excellent 5-year DSSs were observed (Figure 2C). Patients with grade 2 EC and *TP53*-altered or NSMP had 5-year DSSs of 85% to 95% (Figure 2D). Within the external validation cohort of 373 patients, survival outcomes were similarly distributed across all the molecular subgroups, with 5-year DSSs varying between 98% to 100% in low-grade EC and 62% to 100% in high-grade EC (*P* = .02) (eFigure 2 in Supplement 1).

In multivariable analysis of the entire cohort, high-grade (hazard ratio [HR], 4.29; 95% CI, 2.15-8.53; *P* < .001), *TP53*-altered (HR, 1.76; 95% CI, 1.04-2.95; *P* = .03), and FIGO advanced-stage (HR, 4.26; 95% CI, 2.50-7.26; *P* < .001) disease were independently associated with reduced DSS. Among patients with low-grade EC, FIGO advanced stage was independently associated with a reduced DSS, but none of the of molecular subgroups were. However, the number of events was low and the estimated HR’s were of similar magnitude as in the entire cohort (Table 2). Among patients with high-grade EC, only FIGO advanced-stage remained associated as an independent prognostic factor for a reduced DSS (eTable 3 in Supplement 1). Including the diagnostic year in the multivariable Cox regression analyses did not change the results of the Cox regression analyses.

**Table 2.** Cox Regression Univariable and Multivariable Analysis of Disease-Specific Survival in the Entire Cohort and Within Low-Grade EC

Variable	Entire cohort					Low-grade EC				
	Univariable DSS		Multivariable DSS, 73 events		Univariable DSS			Multivariable DSS, 12 events		
	HR (95% CI)	*P* value	HR (95% CI)	*P* value	HR (95% CI)		*P* value	HR (95% CI)	*P* value
Patient age (continuous)	1.04 (1.02-1.07)	.001	1.02 (0.99-1.05)	.08	NA^a^		NA^a^	NA^a^	NA^a^
Grade									
1-2	1 [Reference]	<.001	1 [Reference]	<.001	NA^a^		NA^a^	NA^a^	NA^a^
3	7.70 (4.13-14.35)		4.29 (2.15-8.53)		NA^a^			NA^a^	
Molecular subgroup									
*POLE*-alt	0.17 (0.02-1.27)	.09	0.16 (0.02-1.16)	.07	0.00 (0.00-0.00)		.99	0.00 (0.00-0.00)	.98
MSI	0.45 (0.19-1.11)	.08	0.51 (0.21-1.22)	.13	0.73 (0.15-3.40)		.69	0.65 (0.13-3.02)	.58
*TP53*-alt	4.14 (2.53-6.75)	<.001	1.76 (1.04-2.95)	.03	1.58 (0.19-12.63)		.66	2.94 (0.33-25.83)	.63
NSMP	1 [Reference]	NA	1 [Reference]	NA	1 [Reference]		NA	1 [Reference]	NA
LVSI									
No	1 [Reference]]	<.001	1 [Reference]	.64	1 [Reference]		.30	1 [Reference]	.78
Yes	3.78 (2.37-6.00)		1.13 (0.67-1.88)		2.27 (0.48-10.57)			1.28 (0.24-6.88)	
FIGO									
Stage I-II	1 [Reference]	<.001	1 [Reference]	<.001	1 [Reference]		.01	1 [Reference]	.008
Stage III-IV	7.02 (4.35-11.33)		4.26 (2.50-7.26)		4.57 (1.43-14.56)			5.38 (1.55-18.62)	

Abbreviations: alt, altered; EC, endometrial cancer; FIGO, Federation International of Gynecology and Obstetrics; HR, hazard ratio; LVSI, lymphovascular space invasion; MSI, microsatellite instability; NA, not applicable; NSMP, no specific molecular profile.

^a^ Cox regression analysis within patients with low-grade EC did not include age and grade as variables.

## Discussion

This cohort study assessed whether molecular profiling is associated with outcomes in patients with low-grade EC. Interestingly, patients with low-grade EC had very favorable 5-year DSSs independent of the molecular subgroups compared with patients with high-grade EC. Furthermore, high-grade EC, as well as *TP53*-altered tumors and FIGO advanced-stage disease, were independently associated with decreased DSS. Among patients with low-grade EC, none of the molecular subgroups were independently associated with reduced DSS.

Our study supported previous findings^3,13^ regarding the excellent prognosis for *POLE*-altered EC, good or intermediate prognosis for MSI and NSMP EC, and poor prognosis for *TP53*-altered tumors when analyzing all histological subtypes. Moreover, this study illustrated that the molecular subgroups were mainly discriminative among high-grade EC.^3,8^ To our knowledge, no previous studies have evaluated outcomes for the molecular subgroups within patients with low-grade EC. We analyzed the open access data of Kandoth et al^3^ to validate our results.

Molecular profiling has been proposed to be performed routinely in all patients with EC.^2,17^ However, as most patients with EC are diagnosed with low-grade disease, it is questioned whether this strategy is beneficial and cost-effective. Our data on low-grade EC demonstrate that full molecular profiling may not be necessary (except for screening for Lynch syndrome).^18^ Multivariate analyses did not show any statistically significant association of the molecular subgroups among patients with low-grade EC. However, the number of events was low in this subgroup analysis. Analyzing the HRs, the high HR of *TP53*-altered tumors could still be associated with a reduced DSS in patients with low-grade EC. We question whether this is mainly attributable to grade 2 EC, as shown in the DSS curve of *TP53*-altered tumors within grade 2 EC. Poor interobserver reproducibility is mainly observed within grade 2 and 3 EC; in these patients, the use of immunohistochemical or molecular markers could be recommended, eg, *TP53* genomic or expression analysis in patients with doubtful low-grade (grade 2) EC.^4,8,19,20^ In this way, binary grading (low vs high) with molecular profiling or immunohistochemistry could be optimized with respect to reproducibility.^2^

Molecular profiling is demanding for health care facilities and comes with high costs, which can be especially challenging in low-income countries. Therefore, primary clinical management of EC should be guided based on morphological tumor characteristics, consideration of immunohistochemistry in doubtful cases, and selective molecular profiling in patients with high-grade or advanced-stage disease to guide adjuvant treatment decisions.^21^

To our knowledge, this is the first study to address the prognostic relevance of molecular profiling in low-grade EC. Our study consisted of a large study population, with known lymph node status in FIGO early-stage disease to prevent bias by undiagnosed stage III. Furthermore, our results are comparable with the data of the Cancer Genome Atlas research network.^3^

### Limitations

This study has a few limitations, including those owing its retrospective design. First, differences in the methods between the included studies exist. More than 80% of the cohort was assessed with complete molecular profiling and less than 20% with the immunohistochemistry surrogates of molecular profiling according to the Proactive Molecular Risk Classifier for Endometrial Cancer criteria. However, immunohistochemistry surrogate analysis has been established as a reliable alternative for molecular profiling.^13^ Second, the original diagnosis was used without centralized pathology review; however, slides were from large referral hospitals, and diagnoses were made by expert gynecological pathologists. This makes our study applicable to daily practice. Third, race and ethnicity have not been reported in our study. Although we fully agree that these patients’ race and ethnicity might impact outcomes in several diseases, within Europe they are not routinely documented in patient files.^22^ To evaluate whether race and ethnicity might have impacted our results, we performed additional analyses within the Kandoth et al open access database.^3^ Race was not statistically different between patients with low- vs high-grade EC or between EC-related mortality.^3^ However, in patients with Black race, *TP53*-altered tumors was more frequently present, supporting previous findings of a study by Lu et al^23^ that these women more often were diagnosed with nonendometrioid EC. Therefore, it seems probable that molecular subgroups override the prognostic relevance of race. Fourth, patients were diagnosed between 1994 and 2018, a time spanning more than 24 years, and this could have biased the survival findings because of different treatment strategies over time. Including the diagnostic year in the multivariable Cox regression analyses did not change the results of the Cox regression analyses. Furthermore, although there were significantly more patients with low-grade EC among the excluded patients, the DSS for excluded patients showed similar favorable outcomes for all molecular subgroups within low-grade EC.

## Conclusions

The findings of this cohort study suggest that routine molecular profiling would not be beneficial in patients with low-grade EC due to their excellent prognosis independent of molecular subgroup. Our data demonstrate the importance of primary diagnostic tumor grading and do not support routine molecular profiling in low-grade EC as a cost-effective approach.

## Notes

Supplement 1. **eTable 1.** Baseline Characteristics of the 4 Previous Published Studies and 1 Unpublished Study **eFigure 1.** Study Flowchart **eMethods.** Detailed Information on DNA Analysis, smMIP Design and Library Preparation, Sequencing, and Immunochemistry Analysis **eTable 2.** Baseline Characteristics of the Included vs Excluded Patients **eFigure 2.** Disease-Specific Survival Curves of the Validation Cohort **eTable 3.** Cox Regression Univariable and Multivariable Analysis of Disease-Specific Survival in Patients With High-Grade Disease
eReferences

Supplement 2.
Data Sharing Statement
