# Supplementary material for: Relevance of Molecular Profiling in Patients With Low-Grade Endometrial Cancer
Source: JAMA Netw Open. 2022 Dec 16;5(12):e2247372. doi: 10.1001/jamanetworkopen.2022.47372 (PMC9856566; doi:10.1001/jamanetworkopen.2022.47372)
Supplement: Supplement 1. — eTable 1. Baseline Characteristics of the 4 Previous Published Studies and 1 Unpublished Study eFigure 1. Study Flowchart eMethods. Detailed Information on DNA Analysis, smMIP Design and Library Preparation, Sequencing, and Immunochemistry Analysis eTable 2. Baseline Characteristics of the Included vs Excluded Patients eFigure 2. Disease-Specific Survival Curves of the Validation Cohort eTable 3. Cox Regression Univariable and Multivariable Analysis of Disease-Specific Survival in Patients With High-Grade Disease eReferences [file jamanetwopen-e2247372-s001.pdf]

Supplemental Online Content

Vrede SW, Kasius J, Bulten J, et al. Relevance of molecular profiling in patients with low-grade endometrial cancer. *JAMA Netw Open*. 2022;5(12):e2247372.
doi:10.1001/jamanetworkopen.2022.47372

eTable 1. Baseline Characteristics of the 4 Previous Published Studies and 1 Unpublished Study

eFigure 1. Study Flowchart

eMethods. Detailed Information on DNA Analysis, smMIP Design and Library Preparation, Sequencing, and Immunochemistry Analysis

eTable 2. Baseline Characteristics of the Included vs Excluded Patients

eFigure 2. Disease-Specific Survival Curves of the Validation Cohort

eTable 3. Cox Regression Univariable and Multivariable Analysis of Disease-Specific Survival in Patients With High-Grade Disease

eReferences

This supplemental material has been provided by the authors to give readers additional information about their work.

© 2022 Vrede SW et al. *JAMA Network Open.*

eTable 1. Baseline Characteristics of the 4 Previous Published Studies and 1 Unpublished Study

			Van der Putten et al. 2016[1]	Reijnen et al. 2019[2]	Reijnen et al. 2020[3]	Van Weelden et al. 2020[4]	Reijnen et al. 2022[5]
**Study characteristics**							
N included			265	42	28	28	30
Diagnostic classification based on			Molecular profiling	ProMisE	ProMisE	Molecular profiling	Molecular profiling
Median follow-up (months)			76.0 (0.0-197.0)	41.0 (14.0-87.0)	35.5 (0.0-197.0)	37.5 (3.0-21.0)	34.5 (3.0-168.0)
Demographics			European	European	Dutch	European	Dutch
**Patient characteristic**							
Age (years)			63.0 (34.0-86.0)	66.0 (50.0-82.0)	57.0 (31.0-81.0)	66.0 (45.0-82.0)	67.0 (49.0-77.0)
**Primary treatment**							
Lymph node dissection	No		11 (4.2)	0 (0.0)	0 (0.0)	0 (0.0)	1 (3.3)
	Yes		254 (95.8)	42 (100.0)	28 (100.0)	24 (85.7)	28 (93.3)
		Pelvic	190 (74.8)	0 (0.0)	0 (0.0)	6 (25.0)	18 (64.3)
		Para-aortic	4 (1.6)	0 (0.0)	0 (0.0)	2 (8.3)	7 (25.0)
		Pelvic and para-aortic	45 (17.7)	0 (0.0)	0 (0.0)	9 (37.5)	3 (10.7)
		Unknown which nodes	15 (5.9)	42 (100.0)	28 (100.0)	7 (29.2)	0 (0.0)
	Unknown		0 (0.0)	0 (0.0)	0 (0.0)	4 (14.3)	1 (3.3)
**Final pathologic characteristics**							
Histology	EEC		26 (9.8)	42 (100.0)	20 (71.4)	17 (60.7)	0 (0.0)
	NEEC		239 (90.2)	0 (0.0)	8 (28.6)	11 (39.3)	30 (100.0)
Grade	1-2		183 (69.1)	0 (0.0)	18 (64.3)	8 (28.6)	0 (0.0)
	3		82 (30.9)	42.0 (100.0)	10 (35.7)	20 (71.4)	30 (100.0)
Molecular	POLE-mut		26 (9.8)	3 (7.1)	3 (10.7)	0	1 (3.3)
	MSI		51 (19.2)	15 (35.7)	2 (7.1)	4 (14.3)	6 (20.0)
	TP53-mut		31 (11.7)	9 (21.4)	8 (28.6)	16 (57.1)	8 (26.7)
	NSMP		157 (59.2)	15 (35.7)	15 (53.6)	8 (28.6)	15 (50.0)
MI	<50%		154 (58.1)	4 (9.5)	16 (57.1)	11 (39.3)	12 (40.0)
	>50%		111 (41.9)	36 (85.7)	12 (42.9)	17 (60.7)	18 (60.0)
	Missing		0 (0.0)	2 (4.8)	0 (0.0)	0 (0.0)	0 (0.0)
LVSI	No		219 (82.6)	27 (64.3)	26 (92.9)	14 (50.0)	18 (60.0)
	Yes		46 (17.4)	15 (35.7)	2 (7.1)	14 (50.0)	12 (40.0)
Lymph nodes	N0		231 (87.2)	42 (100.0)	0 (0.0)	16 (57.1)	16 (53.3)
	N1		23 (8.7)	0 (0.0)	0 (0.0)	8 (28.6)	12 (40.0)

© 2022 Vrede SW et al. *JAMA Network Open.*

		Pelvic	14 (60.9)	0 (0.0)	0 (0.0)	4 (50.0)	0 (0.0)
		Para-aortic	4 (17.4)			3 (37.5)	0 (0.0)
		Pelvic and para-aortic	5 (21.7)	0 (0.0)	0 (0.0)	1 (12.5)	0 (0.0)
		Unknown which nodes	0 (0.0)	0 (0.0)	0 (0.0)	0 (0.0)	12 (100.0)
	Nx		6 (2.3)	0 (0.0)	28 (100.0)	4 (14.3)	2 (6.7)
FIGO stage	Early (I-II)		223 (84.2)	42 (100.0)		13 (46.4)	12 (40.0)
	Advanced (III-IV)		42 (15.8)	0 (0.0)	28 (100.0)	15 (53.6)	18 (60.0)
**Adjuvant treatment**							
None			66 (24.9)	12 (28.6)	4 (14.3)	6 (21.4)	9 (30.0)
Radiotherapy			163 (61.5)	25 (59.5)	7 (25.0)	15 (53.6)	15 (50.0)
	EBRT		53 (32.5)	8 (32.0)	0 (0.0)	6 (40.0)	0 (0.0)
	VBT		72 (44.2)	12 (48.0)	0 (0.0)	5 (33.3)	0 (0.0)
	ERBT+VBT		38 (23.3)	5 (20.0)	0 (0.0)	4 (26.7)	0 (0.0)
	Unknown		0 (0.0)	0 (0.0)	7 (100.0)	0 (0.0)	15 (100.0)
Chemotherapy			9 (3.4)	1 (2.4)	14 (50.0)	5 (17.9)	4 (13.3)
Chemoradiation			27 (10.2)	1 (2.4)	2 (7.1)	2 (7.1)	2 (6.7)
Unknown			0 (0.0)	3 (7.1)	1 (3.6)	0 (0.0)	0 (0.0)
**Mortality**							
Recurrence			34 (12.8)	13 (31.0)	9 (32.1)	12 (42.9)	6 (20.0)
Mortality			40 (15.1)	11 (26.2)	12 (42.9)	14 (50.0)	13 (43.3)
EC-related mortality			29 (10.9)	9 (21.4)	9 (32.1)	13 (46.4)	13 (43.3)

Data is presented as No. (%), median (IQR)
Abbreviations: *POLE,* Polymerase epsilon; MSI, Microsatellite instability; *TP53,* Tumor protein 53; NSMP, No-specific molecular profile; EEC, endometrioid endometrial cancer; NEEC, non-endometrioid endometrial cancer; MI, myometrial invasion; LVSI, lymphovascular space invasion; N0, negative lymph nodes, N1, positive lymph nodes; Nx, no information about the lymph nodes; FIGO, Federation International of Gynecology and Obstetrics; EBRT, external beam radition therapy; VBT, vaginal brachytherapy; EC, endometrial cancer.

© 2022 Vrede SW et al. *JAMA Network Open.*

eFigure 1. Study Flowchart

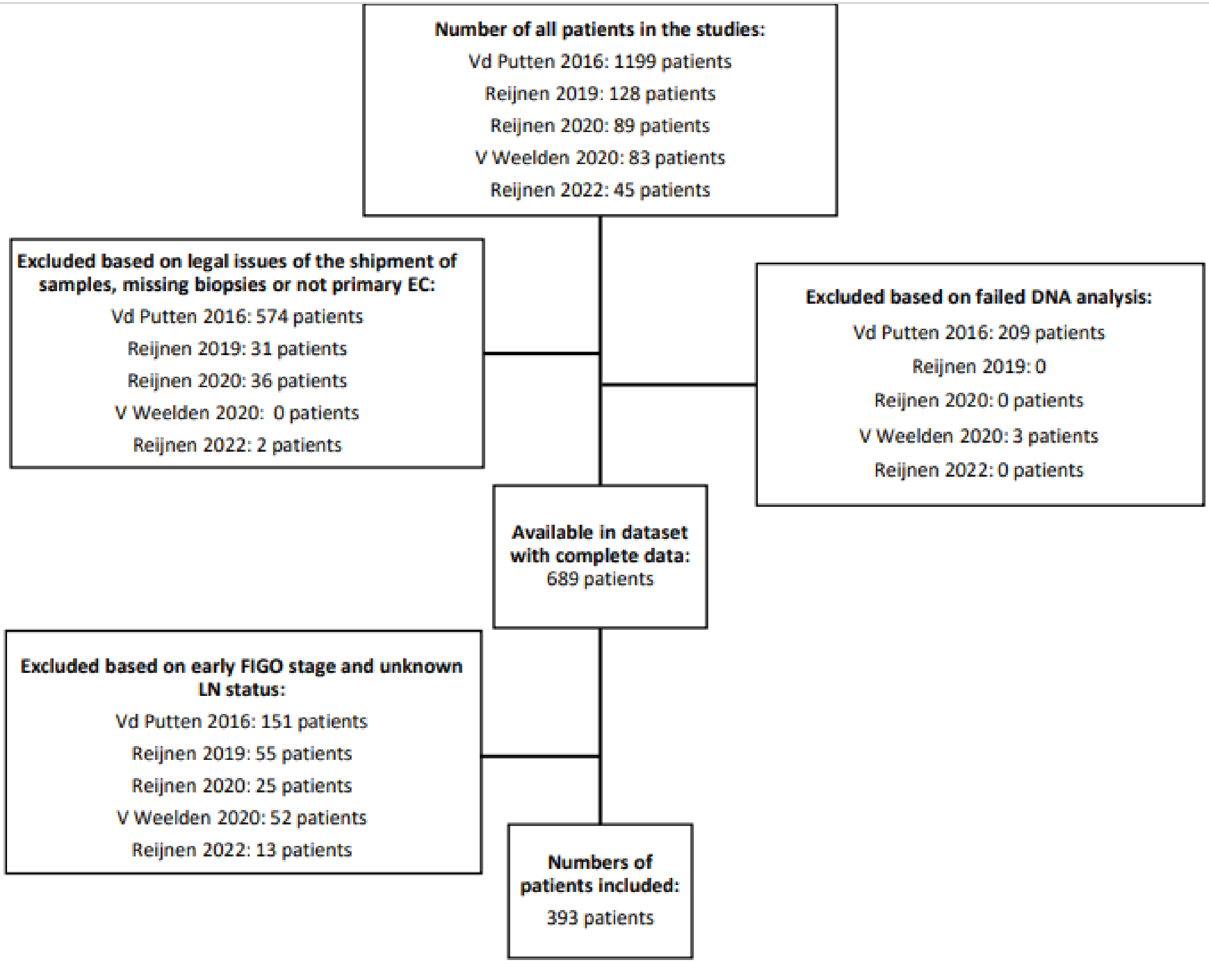

Figure legend: Abbreviations: EC, Endometrial Cancer; LN, Lymph node

© 2022 Vrede SW et al. *JAMA Network Open.*

eMethods. Detailed Information on DNA Analysis, smMIP Design and Library Preparation, Sequencing, and Immunochemistry Analysis

DNA analysis

Representative areas of EC in the surgical specimen were marked and selected for formalin-fixed paraffin-embedded (FFPE) 20 µm thick sections. Slides were cut from these FFPE section and stained with hematoxylin and eosin (H&E). Tumor areas were marked on these slides and the tumor cell percentage was estimated. These specimens were digested overnight at 56°C in TET-lysis buffer (10mmol/L Tris/HCL pH 8.5, 1 mmol/L EDTA pH 8.0, 0.01% Tween-20) with 5% Chelex-100 (Bio-Rad, Hercules, CA) and 0.2% proteinase K, with subsequent inactivation at 95°C for 10 min. After this was centrifugated, the supernatant was transferred into a clean tube. DNA concentration was determined using the Qubit Broad Range Kit (Thermo Fisher Scientific, Waltham, MA).

smMIP design and library preparation

The panel consisted of 10 genes important for EC oncogenesis (ARID1A, CTNNB1, ERBB2, KRAS, MTOR, NRAS, PIK3CA, PTEN, POLE, TP53). The smMIPs were designed in a tilling manner for hotspots in oncogenes and all coding as well as splice site consensus sequences of tumor suppressor genes (TSGs), with preferential targeting of both strand by two independent smMIPs. All the smMIP probes are constructed by an extension and ligation probe arm (40 bp long) with a 112 bp gap and a common backbone sequence for PCR-based library amplification. The backbone and ligation probe arm are connected by means of an 8 bp degenerate sequence (8xN) serving as a Unique Molecular Identifier (UMI, "single-molecule tag"). Following, the smMIP probes were mixed and phosphorylated with 1 µl of T4 polynucleaotide kinase (M0201; New England Biolabs). The molecular ratio between gDNA and smMIPs was set at 1:3,200 for each individual smMIP and the standard genomic DNA input was set at 100ng. A capture mix was made (volume 25 µl) with the phosphorylated smMIP pool, 1 unit of Ampligase DNA ligase (A0110K; EpiBio, Madison, WI) and Ampligase Buffer (A1905B, DNA ligase buffer), 3.2 units of Hemo Klentaq (M0332; New England Biolabs), 8 mmol of dNTPs (28-4065-20/-12/-22/-32; GE Healthcare, Little Chalfont, UK) and 100 ng of genomic DNA in a 20 µl volume. This capture mix was denatured at 95°C for 10 min and subsequently incubated for probe hybridization, extension and ligation for 18hr at 60°C. To perform the exonuclease treatment, Exonuclease 1 (10 units; M0293; New England Biolabs) and III (50 units; M0206; New England Biolabs) and Ampligase Buffer was added to the capture mix after cooling (total of 27 µl). This mix was incubated at 37°C for 45 min, with subsequent inactivation at 95°C for 2 min. From the 27 µl, 20 µl was used for PCR in at total volume of 50 µl including a common forward primer, bar-coded reverse primers, and iProof high fidelity master mix (1725310, Bio-Rad, Veenendaal, the Netherlands). The resulting PCR products were then pooled and purified with 0.8x volume of Agencourt Ampure XP Beads (a63881, Beckman Coulter, Woerden, the Netherlands).

Sequencing

The purified libraries were denatured and diluted to 1.2pmol/l, and then sequenced on a NexSeq500 device (Illumina, San Diego, CA) using the manufacturer's instructions (300 cycles High Output sequencing kit, v2), resulting in 2x150bp paired-end reads. All Bcl files were converted to fastq files and bar-coded reads were then demultiplexed. Single-molecule-directed assembly of the duplicate reads was conducted generating consensus ('unique') reads with the software Sequence Pilot (version 4.4.0; JSI medical system, Ettenheim, Germany).
Variants were annotated as 'malignant', 'likely malignant', 'unknown significance', 'likely benign' and 'benign' using amongst others publicly available databases such as ClinVar (https://www.ncbi.nlm.nih.gov/clinvar/), The Clinical Knowledgebase (CKB, https://ckb.jax.org/), Cancer Genome Interpreter (CGI, https://www.cancergenomeinterpreter.org/home), the Catalog of Somatic Mutations in Cancer (COSMIC, https://cancer.sanger.ac.uk/cosmic), OncoKB (https://www.oncokb.org/), Varsome (https://varsome.com/). The three first categories were taken into consideration and included known activating hotspot mutations for the oncogenes, and missense, nonsense, frameshift and splice site mutations for the included TSGs. Intronic mutations were excluded with exception of splice site sequences. To determine whether sufficient DNA molecules were sequenced to reliably exclude mutation, a cumulative binomial distribution was used for calculating the required unique read depths, above a certain mutant allele frequency with a certainty of >95%.[6] These required read depts were assessed in the context of estimated tumor percentage cells by microscopy.

Immunohistochemical staining

© 2022 Vrede SW et al. *JAMA Network Open.*

For p53 staining, antigen retrieval (30 minutes, pH 6·7) and blocking of endogenous peroxidase with hydrogen peroxide was performed. Subsequently, slides were incubated with p53 antibody (clone DO-7 + BP53-12, dilution 1:600). Powervision+ Poly-HRP was used and visualization was accomplished by using PowerVision DAB substrate solution (Leica Biosystems, Buffalo Grove, IL, US). Counterstaining was performed with hematoxylin, slides were dehydrated and mounted.

For PMS2 and MSH6 staining, antigen retrieval with EnVision FLEX High pH Target Retrieval Solution, and blocking of endogenous peroxidase with hydrogen peroxide was performed. After, slides were incubated with anti-MSH6 (clone EPR3945 1:400, Abcam, Cambridge, UK) or anti-PMS2 (clone A16-4 dilution 1:20, BD Biosciences , San Jose, CA). Incubation was performed with EnVision FLEX and visualized with High pH visualization system. Counterstaining was performed with hematoxylin, slides were dehydrated and mounted.

© 2022 Vrede SW et al. *JAMA Network Open.*

eTable 2. Baseline Characteristics of the Included vs Excluded Patients

		Included N=393	Excluded N=296	*P*
**Patient characteristic**				
Age (years)		63.0 (31.0-82.0)	64.5 (35.0-93.0)	.09
**Pathologic characteristics**				
POLE-mutant		33 (8.4)	14 (4.7)	.001
MSI		78 (19.8)	79 (26.7)	
TP53-mutant		72 (18.3)	29 (9.8)	
NSMP		210 (53.4)	174 (58.8)	
Histology	EEC	318 (80.9)	275 (92.9)	<.001
	NEEC	75 (19.1)	21 (7.1)	
Grade	1-2	209 (53.2)	217 (73.3)	<.001
	3	184 (46.8)	79 (26.7)	
MI	<50%	197 (50.1)	178 (61.0)	.006
	>50%	194 (49.4)	114 (39.0)	
	Unknown	2 (0.5)		
LVSI	No	304 (77.4)	238 (80.4)	.33
	Yes	89 (22.6)	58 (19.6)	
**Adjuvant treatment**				
None		97 (24.7)	148 (50.3)	<.001
Radiotherapy		225 (57.3)	124 (42.2)	
Chemotherapy		33 (8.4)	17 (5.8)	
Chemoradiation		34 (8.7)	5 (1.7)	
Unknown		4 (1.0)		
**Mortality**				
Recurrence		74 (18.8)	38 (12.8)	.013
Mortality		90 (22.9)	55 (18.6)	.17
EC-related mortality		73 (18.6)	26 (8.8)	<.001

Data is presented as No. (%), median (IQR)
Abbreviations: *POLE,* Polymerase epsilon; MSI, Microsatellite instability; *TP53,* Tumor protein 53; NSMP, No-specific molecular profile; EEC, endometrioid endometrial cancer; NEEC, non-endometrioid endometrial cancer; MI, myometrial invasion; LVSI, lymphovascular space invasion; EC, endometrial cancer.

© 2022 Vrede SW et al. *JAMA Network Open.*

eFigure 2. Disease-Specific Survival Curves of the Validation Cohort

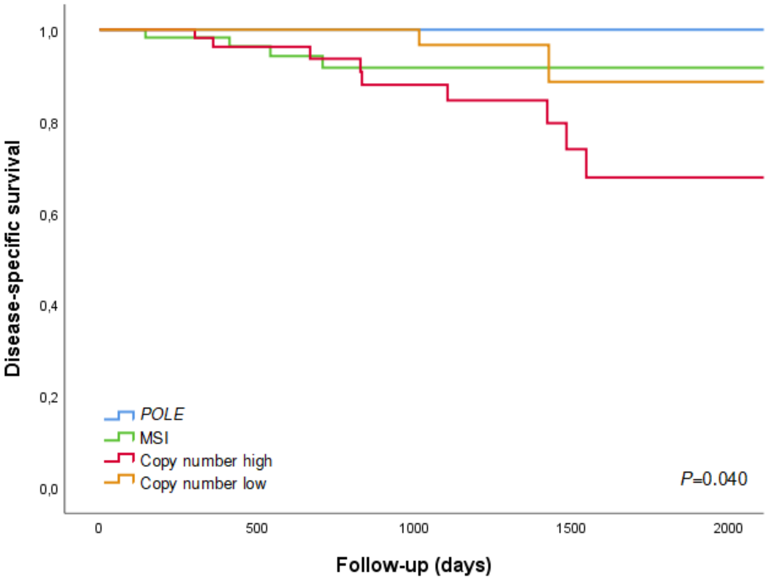

Numbers at risk					
POLE	17	12	9	4	3
MSI	65	46	23	16	11
Copy number high	60	43	28	13	5
Copy number low	90	68	30	10	7

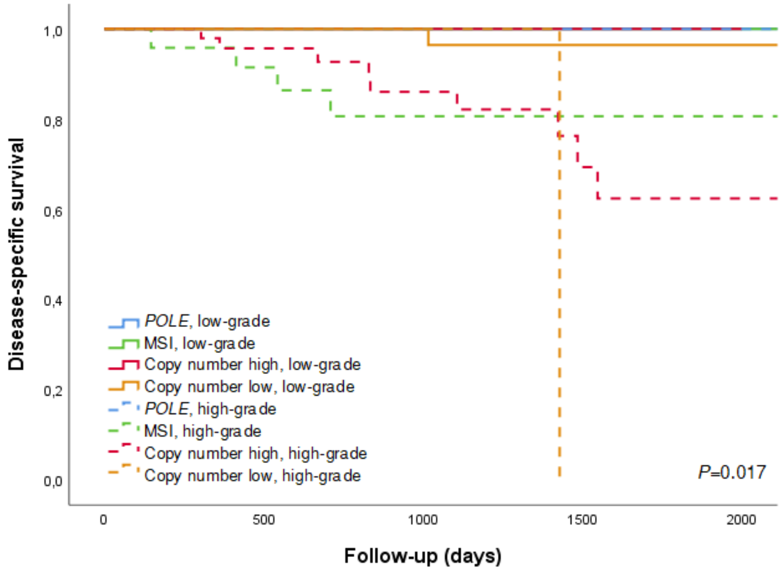

Numbers at risk					
**Low-grade**					
POLE	9	7	5	1	1
MSI	40	27	14	10	7
Copy number high	7	6	4	3	1
Copy number low	84	64	28	10	7
**High-grade**					
POLE	8	5	4	3	2
MSI	25	19	9	6	4
Copy number high	53	37	24	10	4
Copy number low	6	4	2	0	0

Figure legend: A. The 5-years disease-specific survival (DSS) of the molecular subgroups in the entire cohort. B. 5-years DSS of the molecular subgroups and low- versus high-grade endometrial cancer (EC).
Abbreviations: *POLE,* Polymerase epsilon; MSI, Microsatellite instability; *TP53,* Tumor protein 53; NSMP, No-specific molecular profile

© 2022 Vrede SW et al. *JAMA Network Open.*

eTable 3. Cox Regression Univariable and Multivariable Analysis of Disease-Specific Survival in Patients With High-Grade Disease

**Variable**	**Univariable DSS**		**Multivariable DSS**<br>61 events	
	HR (95% CI)	*P* value	HR (95% CI)	*P* value
**Molecular subgroup**				
POLE-mutant	0.15 (0.02-1.09)	.06	0.19 (0.02-1.46)	.12
MSI	0.27 (0.09-0.77)	.02	0.45 (0.16-1.24)	.12
TP53-mutant	1.93 (1.13-3.28)	.02	1.70 (0.99-2.91)	.05
NSMP	1		1	
**LVSI**				
No	1	.002	1	.67
Yes	2.23 (1.34-3.68)		1.12 (0.65-1.92)	
**FIGO**				
Stage I-II	1	<.001	1	<.001
Stage III-IV	5.67 (3.30-9.73)		4.05 (2.24-7.29)	

Abbreviations: DSS, disease-specific survival; HR, hazard ratio; CI, confidence interval; *POLE*, Polymerase epsilon; MSI, Microsatellite instability; *TP53,* Tumor protein; NSMP, No-specific molecular profile, LVSI, lymphovascular space invasion; FIGO, Federation International of Gynecology and Obstetrics.

© 2022 Vrede SW et al. *JAMA Network Open.*

eReferences

1. van der Putten LJ, Visser NC, van de Vijver K, Santacana M, Bronsert P, Bulten J, et al. L1CAM expression in endometrial carcinomas: an ENITEC collaboration study. Br J Cancer. 2016;115(6):716-24.
2. Reijnen C, Küsters-Vandevelde HVN, Prinsen CF, Massuger L, Snijders M, Kommoss S, et al. Mismatch repair deficiency as a predictive marker for response to adjuvant radiotherapy in endometrial cancer. Gynecol Oncol. 2019;154(1):124-30.
3. Reijnen C, Küsters-Vandevelde HVN, Ligtenberg MJL, Bulten J, Oosterwegel M, Snijders M, et al. Molecular profiling identifies synchronous endometrial and ovarian cancers as metastatic endometrial cancer with favorable clinical outcome. Int J Cancer. 2020;147(2):478-89.
4. van Weelden WJ, van der Putten LJM, Inda MA, van Brussel A, Snijders M, Schriever LMM, et al. Oestrogen receptor pathway activity is associated with outcome in endometrial cancer. Br J Cancer. 2020;123(5):785-92.
5. Reijnen CV, S.W.; Draak, R.; Sweegers, S.; Snijders, M.P.L.M.; Gestel van, P.; Eikelenboom, A.; Pijnenborg, J.M.A.;, Bulten, J.; Küsters-Vandevelde, H.V.N. Pure and mixed clear cell carcinoma of the endometrium: a molecular and immunohistochemical analysis study. 2022.
6. Eijkelenboom A, Kamping EJ, Kastner-van Raaij AW, Hendriks-Cornelissen SJ, Neveling K, Kuiper RP, et al. Reliable Next-Generation Sequencing of Formalin-Fixed, Paraffin-Embedded Tissue Using Single Molecule Tags. J Mol Diagn. 2016;18(6):851-63.
