# Supplementary material for: Relevance of Molecular Profiling in Patients With Low-Grade Endometrial Cancer
Source: JAMA Netw Open. 2022 Dec 16;5(12):e2247372. doi: 10.1001/jamanetworkopen.2022.47372 (PMC9856566; doi:10.1001/jamanetworkopen.2022.47372)
Supplement: Supplement 2. — Data Sharing Statement [file jamanetwopen-e2247372-s002.pdf]

Data Sharing Statement

Vrede. Relevance of Molecular Profiling in Patients With Low-Grade Endometrial Cancer. *JAMA Netw Open*. Published December 16, 2022. doi:10.1001/jamanetworkopen.2022.47372

Data
Data available: Yes
Data types: Deidentified participant data
How to access data: Data on request, contact: stephanie.vrede@radboudumc.nl
When available: With publication

Supporting Documents
Document types: None

Additional Information
Who can access the data: Researchers requesting the data
Types of analyses: Any purpose
Mechanisms of data availability: Signed data acces agreement
